# Prevalence and Correlates of Suicidal Distress in Adolescents in a Chronic Pain Service

**DOI:** 10.1002/pne2.70046

**Published:** 2026-09-30

**Authors:** Verena Hinze, Bergljot Gjelsvik, Catherine Crane, Konrad Jacobs

**Affiliations:** ^1^ Department of Psychiatry Warneford Hospital, University of Oxford Oxford UK; ^2^ Department of Psychology University of Oslo Oslo Norway; ^3^ City and South Learning Disability Team Oxford Health NHS Foundation Trust Oxford UK; ^4^ Oxford Centre for Children and Young People in Pain, Children's Hospital Oxford University Hospitals NHS Foundation Trust Oxford UK

**Keywords:** adolescents, burdensomeness, hopelessness, pain, self‐harm, suicidality

## Abstract

Chronic pain is increasingly recognized as an independent correlate of suicidality in adolescence, yet most evidence comes from community‐based studies. This observational, cross‐sectional study (NCT04261114) explored the prevalence and correlates of suicidal distress in a pediatric pain service. Adolescents with chronic pain (*n* = 77; 12–18 years; 79% female; 96% musculoskeletal pain) completed questionnaires on their pain experience and suicidal distress, including perceived burdensomeness, hopelessness, and self‐harm thoughts and behaviors. Self‐harm thoughts and behaviors were common (past two‐week thoughts = 27%, behaviors = 7%; lifetime thoughts = 58%, behaviors = 47%), with prevalence increasing from mid‐adolescence. Perceived burdensomeness and hopelessness were strongly associated with past two‐weeks and lifetime self‐harm thoughts and behaviors (perceived burdensomeness: *r* = 0.31–0.55; hopelessness: *r* = 0.30–0.44). After adjustment for sadness and correction for multiple comparisons, higher perceived burdensomeness was associated with greater social impairment (adjusted risk ratio [aRR] = 1.24 [1.09–1.41]) and pain‐related cognitive intrusions (aRR = 1.25 [1.08–1.44]). Similarly, higher levels of hopelessness were associated with higher daily impairment (aRR = 1.21 [1.08–1.37]), especially social impairment (aRR = 1.32 [1.17–1.48]), and pain‐related cognitive intrusions (aRR = 1.26 [1.12–1.42]). Pain intensity and physical impairment were not associated with suicidal distress outcomes. Exploratory analyses identified additional correlates associated with hopelessness and self‐harm thoughts and behaviors (e.g., constant pain with pain flares, multiple pain locations and widespread pain, greater endorsement of affective and sensory pain descriptors). Suicidal distress is common in pediatric pain services, highlighting the need for routine assessments and integrated psychological support.

**Trial Registration:** The study was prospectively registered on ClinicalTrials.gov (NCT04261114 dated 07/02/2020)

## 1 Introduction

Worldwide, 21% of children and adolescents experience *chronic pain*, defined as persistent/recurrent pain for ≥ 3 months, often presenting as headaches, musculoskeletal pain, abdominal pain, or multisite pain [1]. Prevalence increases during early adolescence from age 12 years onwards, particularly among girls [1, 2]. Adolescence is defined as the developmental period between childhood and adulthood, with an upper age limit of 19 years, consistent with the World Health Organization's definition [3]. This stage is characterized by substantial physical, cognitive, emotional, and social changes that shape lifelong health and well‐being [3]. Although development continues into the third decade of life [4], we adopt this narrower definition because it aligns with the age range typically served by pediatric healthcare services in the UK (e.g., [5]). Chronic pain during adolescence may be particularly consequential, as it can disrupt normative developmental trajectories and compromise the attainment of age‐appropriate developmental goals [6]. Without effective management, chronic pain may persist into adulthood and contribute to disability and distress [6]. Indeed, chronic pain is a leading cause of disability among adolescents globally and often co‐occurs with anxiety and depression [6, 7, 8].

Emerging evidence also links chronic pain to an increased risk of self‐harm and suicidal thoughts and behaviors (hereafter “suicidality”) [9]. Reviews and meta‐analyses consistently show that individuals of all ages with (vs without) physical pain are around twice as likely to report suicidality [9, 10, 11, 12]. Among adults with chronic pain, around 20% report suicidal ideation and 5%–14% report a suicide attempt [11], with similar or higher rates in tertiary pain services (suicidal ideation: 3%–30%; suicide attempt: 6%–23% [9]). Community‐based studies suggest similar concerns in adolescence. In UK secondary schools, 40% of adolescents with persistent/recurrent pain reported suicidality compared with 14% of those without pain [13, 14]. In a Danish Birth Cohort Study, 42% of adolescents with frequent pain at age 11 reported suicidal ideation by age 18 and 7% reported a suicide attempt [15]. These associations persisted after adjustment for key confounders (e.g., childhood trauma, mental health disorders) and were not fully explained by depression, suggesting that chronic pain is an independent correlate of suicidality in adolescence [13, 16].

In adults, suicidality has been associated with several pain‐related factors, including all types of chronic pain, multiple pain conditions, frequent intermittent pain (e.g., migraine), sleep problems, poorer perceived mental health, and psychosocial factors (e.g., hopelessness, perceived burdensomeness, and thwarted belongingness [9]). Perceived burdensomeness, followed by hopelessness, strongly predicted suicidal ideation in adults with chronic pain [17], consistent with leading suicide theories, including the Interpersonal‐Psychological Theory [18, 19] and Three‐Step Theory [20].

However, research examining suicidality among adolescents with chronic pain in clinical settings remains sparse [10]. This is important because the prevalence and correlates of suicidality differ between adolescents and adults [10, 12]. A recent meta‐analysis found that adolescents with pain were at increased risk of self‐harm, whereas adults with pain were at increased risk of suicide attempts and death by suicide [12]. Despite these developmental differences, little is known about the prevalence of suicidality in pediatric pain services and pain‐related correlates of suicidal distress. These may include sensory (e.g., intensity), affective (e.g., unpleasantness), temporal (e.g., pain flares), and functional (e.g., physical, social, and cognitive impairments) dimensions of the pain experience [21]. In this study, *suicidal distress* refers to one pathway leading to suicidal outcomes, ranging from perceived burdensomeness (the belief that one is a burden on others) and hopelessness (general pessimism about the future) to self‐harm thoughts and behaviors [18, 19, 20]. Identifying pain‐related correlates of these precursors aligns with leading suicide theories [18, 19, 20, 22] and may enable earlier identification of at‐risk adolescents before self‐harm thoughts and behaviors develop.

This cross‐sectional study aimed to identify the prevalence of self‐harm thoughts and behaviors among adolescents in a chronic pain service (including age and gender differences) and examine associations between pain‐related correlates and suicidal distress. We hypothesized that greater pain intensity, affective pain endorsement, functional impairment (physical and social), and pain‐related cognitive intrusions would be associated with greater suicidal distress (primarily perceived burdensomeness and secondarily hopelessness and self‐harm thoughts and behaviors).

## 2 Methods

### 2.1 Study Design and Participants

Over a 12‐month period (13/06/2022–12/06/2023), 77 adolescents with chronic pain were recruited from the Oxford Centre for Children and Young People in Pain; a tertiary pediatric pain service at Oxford University Hospitals NHS Foundation Trust (Oxford, United Kingdom). Adolescents were approached by a member of their clinical team during a routine treatment visit, typically at their initial assessment on entry to the pain service, screened for eligibility, and provided with a participant information sheet to review. Participation was entirely voluntary, and adolescents were informed that they could withdraw from the study at any time without providing a reason or affecting their future care. Two eligible adolescents who were approached to participate declined due to concerns that participation might trigger suicidal thoughts. No risks or safeguarding concerns were identified during the study among participants who took part. The multidisciplinary team included psychologists, physiotherapists, occupational therapists, and teachers. Acceptance for assessment in this service reflects the presence of long‐standing, treatment‐resistant chronic pain that significantly impairs functioning. Adolescents with chronic pain were eligible for inclusion if they were aged 12–19 years, fluent in English, and provided written informed consent/assent. Adolescents were excluded if their pain was related to an identifiable acute pathology (e.g., injury) or surgical procedure or if they were deemed unable to safely participate (e.g., acute suicidal crisis), based on clinical judgment. Eligible adolescents who expressed an interest in participating were introduced to a member of the research team, who obtained informed consent before conducting the study procedures. Participants completed a series of questionnaires at the clinic, using JISC Online Surveys, and were compensated for their time. This study received ethical approvals from the South‐Central Oxford B Research Ethics Committee (Ref: 19/SC/0617; date: 23/01/2020). All participants provided informed consent/assent prior to their participation in this study.

### 2.2 Measures

#### 2.2.1 Chronic Pain Experience

The Adolescent Pediatric Pain Tool (APPT [23]) was used to measure participants' typical pain intensity (rating scale: 1 = “no pain” to 5 = “worst possible pain”) and location (15 body areas to identify the number and type of pain locations; Figure S1). We created a categorical “Pain location” variable to identify localized (1 location), multiple (≥ 1 separate locations), and widespread pain (multiple/extensive locations, above and below the waist). A list of 67 pain descriptors was used to assess sensory (e.g., aching), affective (e.g., terrifying), evaluative (e.g., uncontrollable), and temporal characteristics (e.g., constant), by calculating the proportion of words endorsed for each subscale (range = 0–1). The APPT is a valid and reliable multidimensional measure of pain for children and adolescents aged 8–17 years, with evidence of cross‐cultural validity and sensitivity to change during recovery [24, 25].

Participants identified their typical pain trajectory. We created three groups: constant pain (“persistent pain with slight fluctuations”), pain flares (“pain attacks without pain between them”), and constant pain with pain flares (“persistent pain with pain attacks” or “pain attacks with pain between them”).

Participants scored the 9‐item physical and social functioning subscales of the Bath Adolescent Pain Questionnaire (BAPQ [26]), on a 5‐point Likert Scale (0 = “never” to 4 = “always”). Higher subscale scores (range = 0–36) indicate greater physical or social impairments. Summing both subscales created a total score (range = 0–72), with higher scores representing greater daily impairment [26]. The BAPQ has demonstrated good internal consistency, temporal stability, and construct validity, with psychometric evaluations supporting both the overall scale and its subscales in adolescents with chronic pain [26].

To assess pain‐related cognitive intrusions (i.e., attentional interruption, rumination, and lack of disengagement), participants scored the 10‐item Experience of Cognitive Intrusions of Pain Scale (ECIP [27]) on a 7‐point Likert scale (0 = “not at all applicable” to 6 = “highly applicable”). Higher total scores (range = 0–60) indicate greater cognitive intrusions. Previous research identified a one‐component structure of the ECIP with high internal consistency and convergent validity in adolescents aged 11–18 years in a multidisciplinary chronic pain service [27, 28].

#### 2.2.2 Suicidal Distress

For our primary outcome, participants completed the 5‐item perceived burdensomeness subscale of the Interpersonal Needs Questionnaire (INQ‐10 [29, 30]) rating each item on a 7‐point Likert scale (1 = “not at all true for me” to 7 = “very true for me”). Higher scores (range = 5–35) revealed greater perceived burdensomeness. The perceived burdensomeness subscale of the INQ‐10 has demonstrated good construct validity and internal consistency in adolescent samples aged 12–18 years [29, 31].

Secondary outcomes included hopelessness and self‐harm thoughts and behaviors: The Hopelessness Scale for Children (HSC [32]) is a 17‐item true/false measure of hopelessness. Higher total scores (range: 0–17) represent greater hopelessness. The HSC has demonstrated good internal consistency, 10‐week test–retest reliability, and construct validity in adolescents aged 12–17 years [33].

Recent and lifetime self‐harm thoughts were assessed with two yes/no items, adapted from community‐based surveys [13]: “In the past two weeks, have you thought about trying to harm or hurt yourself in some way, even if you would not really do it?” and “Over the whole of your lifetime, have you ever thought about harming or hurting yourself in some way?”

Recent and lifetime self‐harm behaviors were assessed with two yes/no items, adapted from community‐based surveys [13]: “In the past two weeks, have you actually tried to harm or hurt yourself on purpose in some way?” and “Over the whole of your lifetime, have you ever previously harmed or hurt yourself on purpose in some way?”

#### 2.2.3 Demographic and Mental‐Health Covariates

Participants completed standard demographic questions (e.g., age in years, gender, and ethnicity) and scored the 6‐item depression and 7‐item general anxiety subscales of the BAPQ on a 5‐point Likert Scale (0 = “never” to 4 = “always” [26]). Given the item overlap with our outcome measures, we used the item “I feel sad” as a measure of sadness and “I am afraid” as a measure of fear (Table S1).

### 2.3 Statistical Analyses

Statistical analyses were undertaken in R v4.3.3. We used complete‐case analyses, as we had no missing data, except one participant not answering the question on recent self‐harm. If a single item was missing, mean imputation was used to compute total scores. We used descriptive statistics to describe the prevalence of suicidal distress overall and by age and gender.

To explore correlates of suicidal distress, we computed Spearman's rho correlation coefficients and point‐biserial correlations, with 95% confidence intervals [95% CIs], using bias‐corrected and accelerated bootstrapping (*N* = 10 000 samples). We conducted a series of univariable regression analyses. Given the positively skewed continuous data (Figure 1), we investigated whether perceived burdensomeness and hopelessness should be estimated with a gamma distribution. We fitted two unconditional means models using a gamma distribution with log link (R package *glm*()) and ordinary least squares linear regression (R package *lm*()). Since the gamma model provided a better model fit (perceived burdensomeness: linear model: AIC = 558.6, BIC = 563.2; gamma model: AIC = 527.1, BIC = 531.8; hopelessness: linear model: AIC = 425.7, BIC = 430.4; gamma model: AIC = 415.9, BIC = 420.5), we proceeded with the gamma model with log link and report risk ratios [RRs]. To estimate the presence or absence of self‐harm thoughts and behaviors, we used generalized linear models (R package *glm*()) with a binomial distribution with logit link and report odds ratios [ORs]. We mean‐centred our continuous predictors and report model‐based and standardized estimates. To test our hypotheses, we estimated adjusted models. Given the high comorbidity of adolescent chronic pain with depression and anxiety [7], and item overlap between the depression and anxiety scales and our outcome measures (Table S1), we controlled for sadness, and conducted sensitivity analyses, controlling for sadness and fear to avoid multicollinearity and over‐adjustment.

An a priori power calculation was conducted for the continuous outcomes perceived burdensomeness (primary outcome) and hopelessness (secondary outcome). This indicated that a sample size of 77 participants would be sufficient for the planned regression analyses, including two independent variables (predictor: pain variable, covariate: sadness), to detect a medium effect (*f* ^2^ = 0.15) with 80% power (*α* = 0.05), assuming 15% missingness. As the study was not powered to detect effects for the binary outcomes of self‐harm thoughts and behaviors, analyses of these outcomes were treated as exploratory and are intended to inform future research. Hence, for perceived burdensomeness and hopelessness only, *p*‐values were corrected for multiple testing using the Benjamini–Hochberg false discovery rate procedure.

## 3 Results

Of the 77 participants, aged mean (range) 15.4 (12–18 years), most identified as female (79%) and White British/European (91%; Table 1). Sixty (78%) participants reported constant pain with pain flares of medium to high intensity (mean (SD) = 3.55 (0.77), range = 2 (“little pain”)‐5 (“worst possible pain”)). Most participants (57%) reported widespread pain, with a mean (SD) of 5 (3.29) pain locations (range = 1–15). Musculoskeletal pain (96%) was most frequently reported, followed by headaches (57%) and abdominal/pelvic pain (27%; Figure S1).

**TABLE 1:** Participant characteristics.

Demographics and mental health covariates	12–15‐year‐olds (*n* = 36; 47%)	16–18‐year‐olds (*n* = 41; 53%)	Total (*N* = 77; 100%)
Age in years, mean (SD) [range in years]	n/a	n/a	15.4 (1.52) [12–18]
Gender identity, *n* (%)			
Female	28 (78%)	33 (81%)	61 (79%)
Male	6 (17%)	4 (10%)	10 (13%)
Other/prefer not to say	2 (6%)	4 (10%)	6 (8%)
Ethnicity, *n* (%)			
White British/European	34 (94%)	36 (88%)	70 (91%)
Asian	1 (3%)	2 (5%)	3 (4%)
Mixed and other ethnic minorities	1 (3%)	3 (7%)	4 (5%)
Sadness, mean (SD) [range]	2.67 (0.72) [1–4]	2.44 (0.87) [0–4]	2.55 (0.80) [0–4]
Fear, mean (SD) [range]	2.11 (1.17) [0–4]	2.10 (1.07) [0–4]	2.10 (1.11) [0–4]
*Aspects of the pain experience*			
Pain intensity, mean (SD) [range]	3.64 (0.64) [2–5]	3.46 (0.87) [2–5]	3.55 (0.77) [2–5]
Pain patterns, *n* (%)			
Constant pain	5 (14%)	4 (10%)	9 (12%)
Pain flares	4 (11%)	4 (10%)	8 (10%)
Constant pain & pain flares	27 (75%)	33 (80%)	60 (78%)
Pain location, *n* (%)			
Localized pain	3 (8%)	3 (7%)	6 (8%)
Multiple, separate locations	12 (33%)	15 (37%)	27 (35%)
Widespread pain	21 (58%)	23 (56%)	44 (57%)
No of pain locations, mean (SD) [range]	5.11 (3.62) [1–15]	5.22 (3.01) [1–11]	5.17 (3.29) [1–15]
Headache, *n* (%)	21 (58%)	23 (56%)	44 (57%)
Abdominal/Pelvic pain, *n* (%)	12 (33%)	9 (22%)	21 (27%)
Musculoskeletal pain, *n* (%)	33 (92%)	41 (100%)	74 (96%)
Qualitative aspects, mean (SD) [range]			
Proportion of sensory words	0.26 (0.19) [0.03–0.70]	0.29 (0.17) [0.05–0.73]	0.27 (0.18) [0.03–0.73]
Proportion of affective words	0.21 (0.27) [0.00–1.00]	0.19 (0.23) [0.00–1.00]	0.20 (0.25) [0.00–1.00]
Proportion of evaluative words	0.55 (0.32) [0.12–1.00]	0.53 (0.30) [0.00–1.00]	0.54 (0.31) [0.00–1.00]
Proportion of temporal words	0.29 (0.18) [0.00–0.82]	0.30 (0.11) [0.09–0.55]	0.30 (0.15) [0.00–0.82]
Daily impairment, mean (SD) [range]	34.64 (11.30) [14–56]	32.78 (10.91) [11–52]	33.65 (11.06) [11–56]
Physical, mean (SD) [range]	15.75 (7.78) [4–33]	14.32 (7.61) [3–29]	14.99 (7.68) [3–33]
Social, mean (SD) [range]	18.89 (7.08) [4–34]	18.46 (5.48) [5–27]	18.66 (6.24) [4–34]
Cognitive intrusions, mean (SD) [range]	32.42 (15.93) [0–60]	33.71 (13.19) [4–60]	33.10 (14.45) [0–60]
*Suicidal distress*			
Perceived burdensomeness, mean (SD) [range]	12.89 (8.14) [5–30]	13.02 (9.66) [5–34]	12.96 (8.92) [5–34]
Hopelessness, mean (SD) [range]	6.33 (3.67) [1–15]	6.59 (3.89) [1–15]	6.47 (3.76) [1–15]
Self‐harm thoughts, *n* (%)			
Recent (past 2 weeks)	5 (14%)	16 (39%)	21 (27%)
Lifetime	19 (53%)	26 (63%)	45 (58%)
Self‐harm behaviors, *n* (%)			
Recent (past 2 weeks)	3 (9%)^a^	2 (5%)	5 (7%)^a^
Lifetime	17 (47%)	19 (46%)	36 (47%)

*Note:* Possible ranges: Age = 12–19 years; Sadness = 0–4; Fear = 0–4; Pain intensity = 1(“no pain”) to 5 (“worst possible pain”); Number of pain locations = 1–15; Qualitative aspects = 0.00–1.00; Daily impairment = 0–72; Physical impairment = 0–36; Social impairment = 0–36; Cognitive intrusions = 0–60; Perceived burdensomeness = 5–35; Hopelessness = 0–17.

^a^ *n* = 1 missing value.

### 3.1 Prevalence of Suicidal Distress

The mean (SD) of perceived burdensomeness was 12.96 (8.92) and for hopelessness it was 6.47 (3.76).

Recent and lifetime self‐harm thoughts and behaviors were common (past two‐week thoughts: 27%; behaviors: 7%; lifetime thoughts: 58%; behaviors: 47%). Perceived burdensomeness was strongly associated with hopelessness (*r* [95% CI] = 0.69 [0.54–0.79]), self‐harm thoughts (past two‐week thoughts: 0.55 [0.33–0.71]; lifetime thoughts: 0.43 [0.23–0.57]), and self‐harm behaviors (past two‐week self‐harm: 0.31 [−0.01–0.53]; lifetime self‐harm: 0.37 [0.14–0.55]; Table S2). Likewise, hopelessness was strongly associated with self‐harm thoughts (past two‐week thoughts: 0.44 [0.24–0.61]; lifetime thoughts: 0.41 [0.19–0.56]), and self‐harm behaviors (past two‐week self‐harm: 0.30 [0.07–0.55]; lifetime self‐harm: 0.38 [0.16–0.56]; Table S2). In other words, participants with higher (vs. lower) levels of perceived burdensomeness and hopelessness more often reported recent and lifetime self‐harm thoughts and behaviors (Figure 1).

**FIGURE 1:**
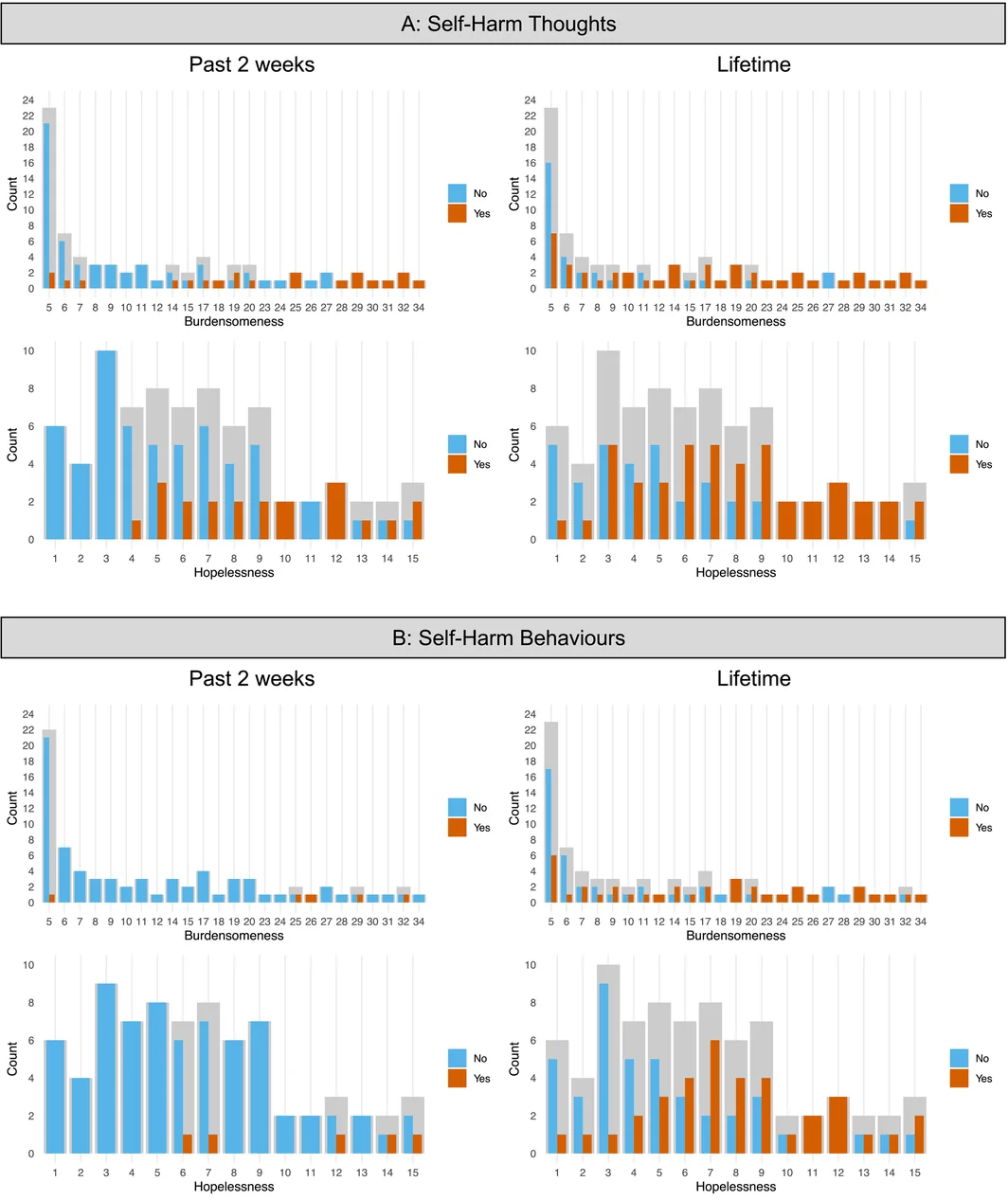
Distribution of hopelessness and perceived burdensomeness scores, overall (gray) and by the presence (orange) and absence (blue) of self‐harm thoughts and behaviors in the past 2 weeks and lifetime.

Figure 2 shows the prevalence of self‐harm thoughts and behaviors by age. Lifetime self‐harm thoughts and behaviors were reported by participants as young as 12 years. Recent self‐harm thoughts were most frequently reported by older adolescents (aged 16 years), whereas recent self‐harm behaviors were uncommon. Compared to 16‐to‐18‐year‐olds (53%), 12‐to‐15‐year‐olds (47%) more often identified as male (17% vs. 10%) and less frequently as “other/prefer not to say” (6% vs. 10%), and had higher rates of abdominal/pelvic pain (33% vs. 22%) and lower rates of musculoskeletal pain (92% vs. 100%), recent self‐harm thoughts (14% vs. 39%), and lifetime self‐harm thoughts (53% vs. 63%; Table 1).

**FIGURE 2:**
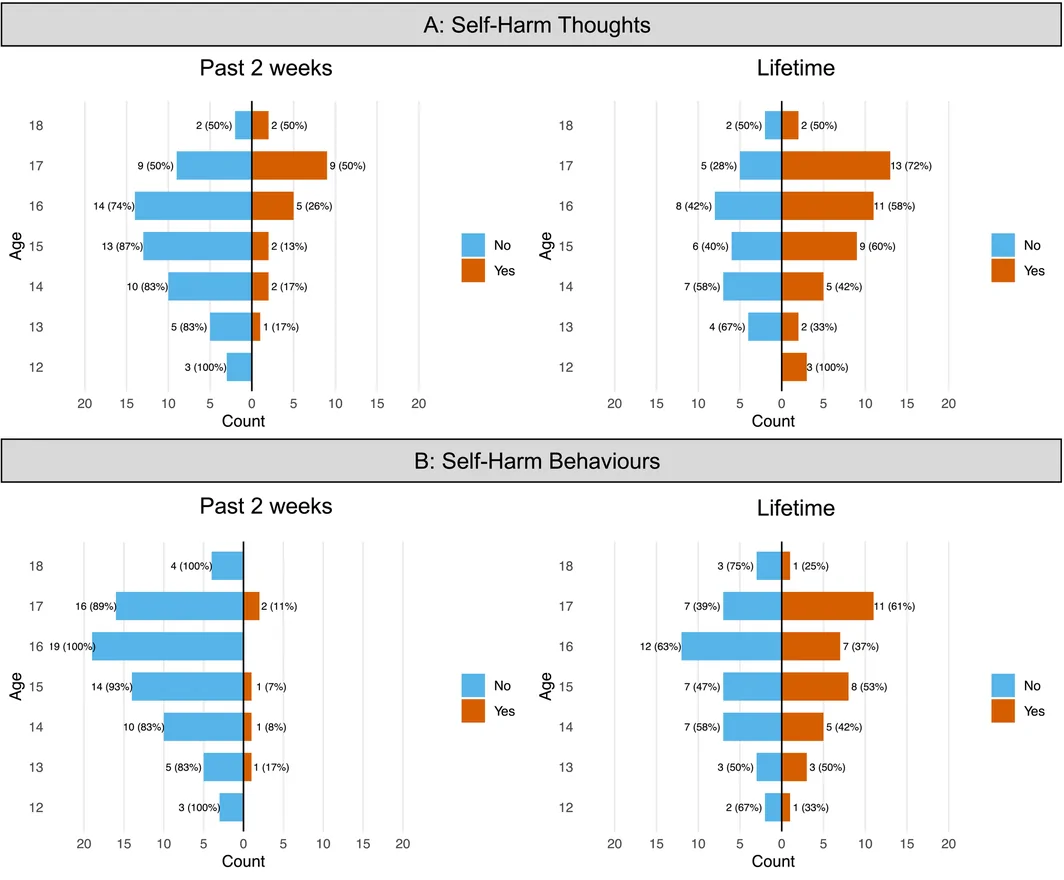
Prevalence of self‐harm thoughts and behaviors by age.

As shown in Figure 3, the prevalence of self‐harm thoughts and behaviors varied by gender. Adolescents, who selected “other/prefer not to say” to the gender question (*n* = 6) reported the highest prevalence (past two‐week thoughts: 50%; behaviors: 33%; lifetime thoughts and behaviors: both 83%), followed by females (*n* = 61; past two‐week thoughts: 28%; behaviors: 5%; lifetime thoughts: 57%; behaviors: 46%) and males (*n* = 10; past two‐week thoughts: 10%; behaviors: 0%; lifetime thoughts: 50%; behaviors: 30%). Compared to boys, girls and adolescents, who selected “other/prefer not to say” to the gender question, were older and reported a higher number of pain locations, proportions of sensory, affective, and evaluative pain descriptors, and greater daily impairment (especially physical), cognitive intrusions, and rates of perceived burdensomeness and hopelessness (Table S3).

**FIGURE 3:**
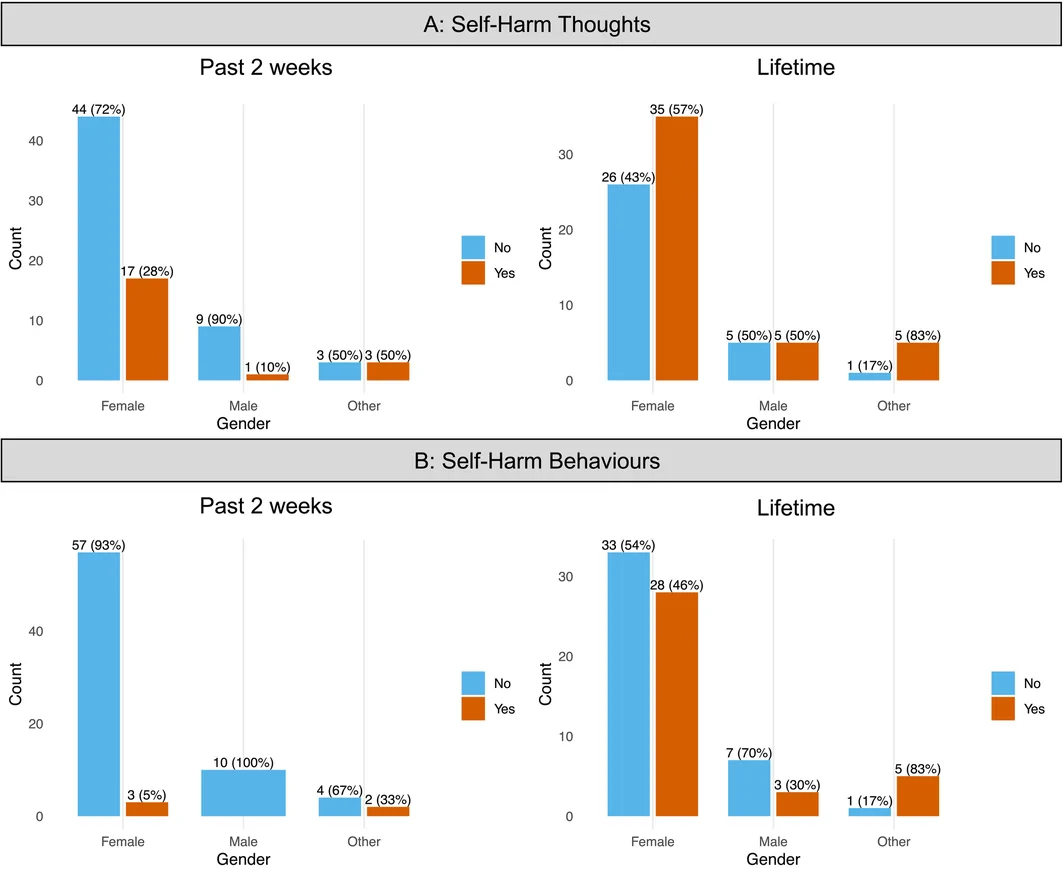
Prevalence of self‐harm thoughts and behaviors by gender.

### 3.2 Correlates of Suicidal Distress

Table S2 shows the correlation coefficients for associations between pain‐related correlates, mental health covariates (i.e., sadness and fear), and suicidal distress. Sadness was strongly associated with all outcomes except recent self‐harm (*r* [95% CI] burdensomeness: 0.47 [0.27–0.63] *p* < 0.001; hopelessness: 0.53 [0.34–0.66] *p* < 0.001; past two‐week thoughts: 0.31 [0.13–0.47] *p* = 0.006; lifetime thoughts: 0.31 [0.08–0.50] *p* = 0.006; past two‐week behavior: 0.21 [0.10–0.42] *p* = 0.062; lifetime behavior: 0.34 [0.13–0.51] *p* = 0.003). Likewise, fear was strongly associated with all outcomes except recent self‐harm (r [95% CI] burdensomeness: 0.41 [0.20–0.58] *p* < 0.001; hopelessness: 0.46 [0.24–0.65] *p* < 0.001; past two‐week thoughts: 0.31 [0.11–0.49] *p* = 0.006; lifetime thoughts: 0.25 [0.02–0.45] *p* = 0.030; past two‐week behavior: 0.12 [−0.11 to 0.33] *p* = 0.322; lifetime behavior: 0.31 [0.10–0.50] *p* = 0.005).

Table 2 presents the standardized coefficients of the regression analyses, adjusted for sadness (Table S4 presents the unadjusted analyses). For perceived burdensomeness and hopelessness, *p*‐values were corrected for multiple testing, whereas analyses of self‐harm thoughts and behaviors were treated as exploratory to inform future research. Consistent with our hypotheses, higher perceived burdensomeness was associated with greater social impairment (aRR = 1.24 [1.09–1.41]) and cognitive intrusions (aRR = 1.25 [1.08–1.44]). Similarly, higher levels of hopelessness were associated with higher daily impairment (aRR = 1.21 [1.08–1.37]), especially social impairment (aRR = 1.32 [1.17–1.48]), and cognitive intrusions (aRR = 1.26 [1.12–1.42]). In contrast to our hypotheses, greater pain intensity, a higher proportion of affective pain descriptors, and greater physical impairment were not associated with perceived burdensomeness and hopelessness.

**TABLE 2:** Regression analyses, adjusted for sadness, using standardized estimates.

Covariates	Perceived burdensomeness			Hopelessness			Self‐harm thoughts (past 2 weeks)^a^			Self‐harm thoughts (lifetime)^a^			Self‐harm behaviors (past 2 weeks)^a^			Self‐harm behaviors (lifetime)^a^		
	aRR	95% CI	*p*	aRR	95% CI	*p*	aOR	95% CI	*p*	aOR	95% CI	*p*	aOR	95% CI	*p*	aOR	95% CI	*p*
Age	1.07	[0.92–1.23]	0.350	1.04	[0.92–1.18]	0.507	2.47	[1.34–5.10]	**0.007**	1.30	[0.80–2.16]	0.301	0.86	[0.36–2.12]	0.727	1.14	[0.70–1.87]	0.594
Gender: Female	Ref	Ref	Ref	Ref	Ref	Ref	Ref	Ref	Ref	Ref	Ref	Ref	Ref	Ref	Ref	Ref	Ref	Ref
Male	0.67	[0.46–1.01]	**0.043**	0.80	[0.56–1.16]	0.208	0.31	[0.02–1.93]	0.291	0.74	[0.18–3.01]	0.664	0.00	[0.00–0.00]	0.996	0.51	[0.10–2.12]	0.374
Other	1.46	[0.92–2.46]	0.126	1.09	[0.71–1.77]	0.696	2.19	[0.36–13.4]	0.374	2.89	[0.41–58.1]	0.354	11.0	[1.09–121.2]	**0.035**	4.76	[0.68–95.85]	0.172
Anxiety	1.15	[0.98–1.35]	0.087	1.18	[1.03–1.36]	**0.018**	1.70	[0.90–3.34]	0.105	1.70	[1.06–2.86]	**0.034**	1.04	[0.33–3.31]	0.945	1.53	[0.87–2.77]	0.143
*Hypothesized effects*																		
Pain intensity	0.96	[0.83–1.10]	0.547	1.01	[0.89–1.14]	0.934	0.73	[0.41–1.27]	0.269	0.46	[0.25–0.80]	**0.009**	1.20	[0.46–3.37]	0.718	0.68	[0.39–1.12]	0.140
% affective words	1.10	[0.96–1.26]	0.211	1.09	[0.96–1.24]	0.196	1.13	[0.66–1.93]	0.651	1.45	[0.83–2.81]	0.230	2.85	[1.26–7.69]	**0.018**	1.51	[0.88–2.78]	0.153
Daily impairment	1.12	[0.99–1.28]	0.090	1.21	[1.08–1.37]	0.001*	0.94	[0.53–1.65]	0.819	1.09	[0.66–1.81]	0.739	3.55	[1.14–15.50]	**0.049**	1.35	[0.82–2.30]	0.247
Physical impact	1.01	[0.88–1.16]	0.931	1.08	[0.95–1.22]	0.220	0.76	[0.41–1.33]	0.355	0.81	[0.50–1.31]	0.399	2.38	[0.86–7.97]	0.111	1.01	[0.62–1.64]	0.960
Social impact	1.24	[1.09–1.41]	0.002*	1.32	[1.17–1.48]	< 0.001*	1.29	[0.70–2.44]	0.413	1.59	[0.94–2.79]	0.090	4.10	[1.10–21.53]	0.056	1.79	[1.04–3.25]	**0.043**
Intrusions	1.25	[1.08–1.44]	0.002*	1.26	[1.12–1.42]	< 0.001*	1.42	[0.77–2.69]	0.268	1.24	[0.73–2.13]	0.434	3.37	[0.99–16.91]	0.084	1.30	[0.76–2.27]	0.333
*Exploratory effects*																		
No of pain locations	1.05	[0.91–1.21]	0.507	1.12	[0.99–1.27]	0.057	0.93	[0.54–1.58]	0.802	1.27	[0.78–2.15]	0.344	3.21	[1.28–10.80]	**0.024**	1.72	[1.04–2.97]	**0.042**
Localized pain	Ref	Ref	Ref	Ref	Ref	Ref	Ref	Ref	Ref	Ref	Ref	Ref	Ref	Ref	Ref	Ref	Ref	Ref
Multiple locations	1.25	[0.71–2.07]	0.417	1.87	[1.16–2.88]	0.007*	2.94	[0.34–64.1]	0.376	0.79	[0.11–5.49]	0.809	0.80	[0.00–0.00]	0.999	0.73	[0.10–6.50]	0.755
Widespread pain	1.24	[0.71–2.02]	0.416	1.84	[1.15–2.80]	0.007*	0.99	[0.12–21.2]	0.994	1.41	[0.20–9.32]	0.715	0.00	[0.00–0.00]	0.998	2.25	[0.34–18.86]	0.405
Constant & flares	Ref	Ref	Ref	Ref	Ref	Ref	Ref	Ref	Ref	Ref	Ref	Ref	Ref	Ref	Ref	Ref	Ref	Ref
Constant pain	0.78	[0.52–1.22]	0.229	0.64	[0.45–0.92]	0.012*	1.70	[0.30–8.53]	0.518	1.50	[0.31–8.07]	0.619	2.96	[0.13–29.76]	0.385	1.24	[0.26–6.13]	0.782
Pain flares	0.56	[0.37–0.90]	**0.010**	0.69	[0.48–1.02]	0.054	0.00	[0.00–0.00]	0.990	5.14	[0.89–48.15]	0.095	0.00	[0.00–0.00]	0.995	1.12	[0.20–5.80]	0.890
% sensory words	1.11	[0.97–1.28]	0.137	1.14	[1.01–1.28]	**0.032**	1.35	[0.79–2.28]	0.261	1.25	[0.76–2.13]	0.381	3.21	[1.29–10.33]	**0.021**	1.47	[0.90–2.49]	0.133
% evaluative words	1.02	[0.88–1.17]	0.829	1.04	[0.91–1.18]	0.603	1.07	[0.60–1.92]	0.811	1.28	[0.76–2.16]	0.357	3.04	[0.88–19.71]	0.132	1.37	[0.82–2.32]	0.231

*Note:* All continuous predictors were mean‐centred. An a priori power calculation was conducted for the continuous outcomes perceived burdensomeness (primary outcome) and hopelessness (secondary outcome). This indicated that a sample size of 77 participants would be sufficient for the planned regression analyses with two independent variables (predictor: pain variable, covariate: sadness) to detect a medium effect (*f* ^2^ = 0.15) with 80% power (*α* = 0.05), assuming 15% missingness. As the study was not powered to detect effects for the binary outcomes of self‐harm thoughts and behaviors, these analyses were treated as exploratory and are intended to inform future research. Statistically significant associations are highlighted in **bold**. For perceived burdensomeness and hopelessness only, *p*‐values for both hypothesized and exploratory effects were corrected for multiple testing using the Benjamini–Hochberg false discovery rate procedure. An asterisk (*) denotes results that remained significant after correction.

^a^ Due to small numbers, confidence intervals are unstable and estimates should be interpreted with caution.

Additional exploratory analyses, adjusted for sadness and corrected for multiple comparisons (Table 2), revealed a lower risk of hopelessness for participants with constant pain, compared to constant pain and pain flares (aRR = 0.64 [0.45–0.92]). A higher risk of hopelessness was observed in participants with pain in multiple locations (aRR = 1.87 [1.16–2.88]) and widespread pain (aRR = 1.84 [1.15–2.80]), compared to localized pain. Additional associations were revealed for perceived burdensomeness and hopelessness, but these associations did not persist following multiple comparison correction (Table 2).

Additional associations were observed for the exploratory outcomes of self‐harm thoughts and behaviors, after adjustment for sadness (Table 2). Higher pain intensity was associated with lower odds of lifetime self‐harm thoughts (aOR = 0.46 [0.25–0.80]). Self‐harm behaviors in the past two weeks were associated with greater daily impairment (aOR = 3.55 [1.14–15.50]), a higher proportion of affective pain descriptors (aOR = 2.85 [1.26–7.69]) and sensory pain descriptors (aOR = 3.21 [1.29–10.33]), as well as a higher number of pain locations (aOR = 3.21 [1.28–10.80]). Lifetime self‐harm behaviors were associated with greater social impairment (aOR = 1.79 [1.04–3.25]) and a higher number of pain locations (aOR = 1.72 [1.04–2.97]).

As fear was associated with hopelessness (aRR = 1.18 [1.03–1.36]) and lifetime self‐harm thoughts (aOR = 1.70 [1.06–2.86]), after adjustment for sadness (Table 2), we conducted sensitivity analyses, controlling for both sadness and fear, revealing similar results (Table S5).

## 4 Discussion

Chronic pain is increasingly recognized as an independent correlate of suicidality in adolescence, yet most evidence comes from community‐based studies and clinical studies remain sparse [10]. To our knowledge, this is the first study to identify the prevalence and correlates of suicidal distress in a UK pediatric pain service. Approximately one in two adolescents reported lifetime self‐harm thoughts and/or behaviors and up to one in four in the past two weeks. Perceived burdensomeness and hopelessness were strongly associated with self‐harm thoughts and behaviors, reinforcing their role as precursors to self‐harm. Sadness and fear were strongly associated with all indicators of suicidal distress, except recent self‐harm. After adjustment for sadness and multiple comparison correction, social impairment and pain‐related cognitive intrusions were associated with perceived burdensomeness and hopelessness, while greater daily impairment was associated with hopelessness specifically. Exploratory analyses revealed additional correlates of hopelessness and self‐harm thoughts and behaviors. Contrary to our hypotheses, pain intensity and physical impairment were not associated with perceived burdensomeness or hopelessness.

The prevalence of self‐harm thoughts and behaviors in the general adolescent population is well‐established. Between 14% and 23% report lifetime self‐harm/suicidal thoughts and 5%–16% engage in self‐harm, particularly girls [34]. In community samples of adolescents with pain, approximately 40% report self‐harm/suicidal thoughts and 7% report self‐harm/suicidal behaviors [13, 15]. Our findings indicate even higher rates in a UK tertiary pain service: 47%–58% of adolescents with chronic pain reported lifetime self‐harm thoughts and behaviors, and 7%–27% reported recent self‐harm thoughts and behaviors. These rates are consistent with reports from US tertiary pain services (recent suicidal ideation: 20%–35% adolescents [35, 36]) and are comparable to adult tertiary pain services (recent suicidal ideation: 3%–30%; lifetime suicide attempt: 6%–23%) [9]. Together, these rates emphasize the need for early risk identification and support in pediatric pain services.

Lifetime self‐harm thoughts and behaviors were reported by adolescents as young as 12 years, indicating pervasiveness across age groups in this service. Although recent self‐harm behaviors were relatively uncommon, recent self‐harm thoughts were common during mid‐adolescence. Adolescents, responding with “other/prefer not to say” to the gender question, reported the highest prevalence, with nearly all (83%) reporting lifetime self‐harm thoughts or behaviors, followed by females and males. Findings align with meta‐analytic evidence showing increases in suicidality during mid‐adolescence and disproportionally higher rates among non‐binary youth and females, possibly reflecting greater exposure to psychosocial risk factors [34, 37].

Consistent with leading suicide theories (the Interpersonal‐Psychological Theory [18, 19] and Three‐Step Theory [20]), perceived burdensomeness and hopelessness were strongly associated with self‐harm thoughts and behaviors, extending evidence from adults with chronic pain [17] to adolescents. Moreover, sadness and fear were strongly associated with all indicators of suicidal distress, apart from recent self‐harm, likely due to its low prevalence. Although adolescents with co‐occurring mental health difficulties may represent a particularly high‐risk subgroup, self‐harm thoughts and behaviors were not confined to those with the most severe depressive symptoms. These results align with evidence that depressive symptoms partially mediate, rather than moderate, the relationship between pain and suicidality in adolescents [13, 15, 36]. Together, these findings support routine biopsychosocial assessments of suicidal distress early in the diagnostic process for all young people with chronic pain, irrespective of depressive symptom severity. While most adolescents with self‐harm thoughts or behaviors will not die by suicide and the motivations, methods, and functions of self‐harm remain unclear, the high prevalence of suicidal distress reflects substantial suffering warranting clinical attention in its own right [38].

Pain‐related social impairment emerged as a salient correlate. Consistent with our hypotheses, greater daily, especially social, impairment was associated with higher perceived burdensomeness and hopelessness, and exploratory analyses revealed similar associations with lifetime and recent self‐harm behaviors. Pain‐related social impairment reflects the inability of spending time with friends and participating in valued social activities, which may contribute to feelings of disconnection and thwarted belonging, which are important precursors to self‐harm/suicidal thoughts [18, 19, 20].

Consistent with our hypotheses, pain‐related cognitive intrusions (e.g., attentional interruption, rumination, lack of disengagement) were associated with perceived burdensomeness and hopelessness. This lack of cognitive escape from pain may contribute to internal entrapment and the “tunnel vision” often observed in a suicidal crisis, where suicide is perceived as the only possible escape [22]. Similarly, associations between recent self‐harm behaviors and greater endorsement of affective and sensory pain descriptors may reflect stronger self‐pain enmeshment (viewing pain as central to one's present and future self) and more elaborate pain representations during periods of suicidal distress. Previous research suggests that stronger self‐pain enmeshment is associated with greater distress [39]. To our knowledge, this is the first study to implicate pain‐related cognitive intrusions and elaborate pain representations in suicidal distress among adolescents with chronic pain. Longitudinal research is needed to examine these processes over time.

Exploratory findings revealed a higher risk of hopelessness among adolescents with constant pain and pain flares, and with pain in multiple locations and widespread. Similarly, adolescents reporting lifetime and recent self‐harm behaviors were more likely to report more pain locations. Findings align with a Danish population‐based study, showing that adolescents with 3+ pain sites at age 11 years were more likely to report lifetime suicidal ideation and behaviors [15]. Pain unpredictability and spreading to multiple locations may amplify perceptions of uncontrollability and diminish expectations of improvements, increasing vulnerability to hopelessness and self‐harm. This interpretation requires longitudinal investigation.

In contrast to our hypotheses, pain intensity and physical impairment were not associated with suicidal distress, aside from an exploratory inverse association between pain intensity and lifetime self‐harm thoughts, which requires replication in larger samples. Evidence on the relationship between pain intensity or physical impairment and suicidality is mixed, with some studies finding weak or inconsistent associations once psychosocial factors are considered [9]. This finding aligns with the broader literature highlighting the multidimensional nature of pediatric chronic pain, in which pain intensity represents only one aspect of the pain experience [6, 40]. Previous research has shown that adolescents with co‐occurring chronic pain and mental health symptoms report significantly poorer social functioning than those with only pain, even after controlling for pain intensity [41]. Together, these findings suggest that severe distress may be more closely associated with the psychosocial consequences of chronic pain than with pain intensity itself [40]. One possible explanation is that, as more time is spent living with chronic pain, suicidal distress may become less closely related to the physical symptoms of pain and more closely related to the psychological and social burden of living with a debilitating condition [9]. Alternatively, pain intensity and physical disability may demonstrate ceiling effects in clinical samples, reducing their ability to discriminate between adolescents at differing levels of suicidal distress [9]. Further research is needed to clarify these explanations.

### 4.1 Clinical Implications

Findings support routine assessments of self‐harm thoughts and behaviors in pediatric pain services and align with the World Health Organization's recommendation to assess suicide risk in individuals aged 10+ years with chronic pain [42]. As adolescents with pain and suicidality are more likely to use healthcare services than those with either condition alone, routine assessments offer vital opportunities for support [14].

Although evidence‐based interventions for pediatric pain and self‐harm are emerging [43, 44], psychological interventions for chronic pain primarily target functional impairment [43] and may not adequately address suicidal distress. Fragmentation between pain and mental health services may further contribute to unmet needs. An integrated approach is needed to address the complex interplay between chronic pain and suicidal distress. This should include supportive attention to psychological wellbeing when adolescents first present with pain to services, routine assessments of suicidal distress, and structured pathways for managing suicidal distress, including closer collaboration with mental health services. Evidence‐based approaches such as safety planning, cognitive‐behavioral therapy, and dialectical behavior therapy for adolescents may be promising, given their focus on cognitive intrusions, emotional regulation, and interpersonal skills [44].

### 4.2 Strengths and Limitations

To our knowledge, this is the first study to identify the prevalence and correlates of suicidal distress in a UK pediatric pain service. Strengths include the clinical setting and explicit examination of both lifetime and recent self‐harm thoughts and behaviors, alongside age and gender differences, and theory‐driven precursors (perceived burdensomeness and hopelessness) that are not routinely assessed in pediatric pain services. The detailed multidimensional assessment of pain (sensory, affective, cognitive, social, and temporal dimensions) enabled identification of risk factors (e.g., constant pain and pain flares in multiple locations or widespread) and potentially modifiable treatment targets (e.g., pain‐related social impairment and cognitive intrusions).

Limitations include the predominantly female and White British/European sample. Whilst this reflects service demographics and aligns with higher rates of chronic pain and suicidality among adolescent girls compared to boys [1, 34], generalisability to more diverse populations (e.g., gender identities and ethnicities) or to adolescents managed exclusively in primary or secondary care is limited. Given the small number of male participants and participants who selected “other/prefer not to say” in response to the gender question, these findings should be interpreted with caution and replicated in larger samples. As the present study focused on adolescents receiving care in pediatric pain services, future research should extend this work to older adolescents and young adults, particularly those transitioning to adult healthcare services, to understand how the transition between pediatric and adult healthcare services influences suicidal distress, its correlates, and opportunities for prevention and intervention. The cross‐sectional design limits conclusions about directionality, and relationships are likely complex and bidirectional. Adolescents with pre‐existing vulnerabilities to suicidal distress may be more likely to experience pain persistence, worsening, and impairments in psychosocial functioning. Furthermore, they may be less likely to respond to treatments offered in primary or secondary care, leading to referral to tertiary services. Longitudinal studies are needed to track trajectories from first presentation in primary care to clarify the timing and mechanisms underlying the emergence of suicidal distress and chronic pain. Finally, wide confidence intervals for some binary outcomes indicate imprecision and require replication in larger samples.

## 5 Conclusion

Self‐harm thoughts and behaviors are common among adolescents in pediatric pain services, with rates increasing during mid‐adolescence. Beyond prevalence, findings identify clinically relevant psychological and pain‐related correlates of suicidal distress, including perceived burdensomeness, hopelessness, pain‐related social impairment and cognitive intrusions, constant pain with pain flares in multiple locations and widespread, and greater endorsement of affective and sensory pain descriptors. Integrated physical and mental healthcare within pain services is paramount, including routine assessments of suicidal distress alongside evaluation of modifiable psychological and social risk factors with strong collaboration between pain and mental health services to ensure timely and appropriate support.

## Author Contributions

V.H. secured the funding and—as chief investigator—had overall responsibility for the management of the study. V.H. developed the study protocol with input from C.C., B.G., and K.J. K.J. supported the recruitment of patients and V.H. collected the data. V.H. had full access to all the data in the study, performed the analyses, and drafted the manuscript. All authors read and approved the final manuscript and accept responsibility for the integrity of the data, the accuracy of the data analysis, and the decision to submit for publication.

## Funding

This study was funded by the Stiftung Oskar‐Helene‐Heim through a postdoctoral research fellowship awarded to V.H. Furthermore, V.H. is funded by the Medical Research Foundation and Arthritis UK (MRF‐FEL‐CAMPCF‐23‐105; Arthritis UK: 23390). The views expressed are those of the author(s) and not necessarily those of the funders. The research funders had no role in the study design, collection, analysis, and interpretation of data, writing of the report, and in the decision to submit the paper for publication. Authors were not precluded from accessing data in the study, and they accept responsibility to submit for publication.

## Ethics Statement

This study was performed in accordance with the ethical standards as laid down in the 1964 Declaration of Helsinki and its later amendments. The study received ethical approvals from the South‐Central Oxford B Research Ethics Committee (Ref: 19/SC/0617; date: 23/01/2020).

## Consent

Informed consent/assent was obtained from all participants included in the study.

## Conflicts of Interest

The authors declare no conflicts of interest.

## Supporting information


**Figure S1:** Pain body map.
**Table S1:** Spearman's rho correlation coefficients between depression and anxiety item scores and perceived burdensomeness and hopelessness, respectively.
**Table S2:** Correlation coefficients for relationships between aspects of the chronic pain experience and suicidal distress.
**Table S3:** Participant characteristics by gender.
**Table S4:** Unadjusted univariable regression analyses.
**Table S5:** Regression analyses, adjusted for sadness and fear, using standardized estimates.

## Data Availability

Deidentified participant data are available upon reasonable request, by contacting the corresponding author. Syntax files related to the current study are available on the Open Science Framework (project title: “Prevalence and Correlates of Suicidal Distress in Adolescents in a Chronic Pain Service”).
