# Supplementary material for: Prevalence and Correlates of Suicidal Distress in Adolescents in a Chronic Pain Service
Source: Paediatr Neonatal Pain. 2026 Sep 30;8(4):e70046. doi: 10.1002/pne2.70046 (PMC13624701; doi:10.1002/pne2.70046)
Supplement: Supplementary file 1 [file PNE2-8-e70046-s001.docx]

**Supplementary Materials**

**Title: Prevalence and Correlates of Suicidal Distress in**

**Adolescents in a Chronic Pain Service**

Short title: Suicidal Distress in a Paediatric Pain Service

Verena Hinze, DPhil,^1^ Bergljot Gjelsvik, PhD,^1,2^

Catherine Crane, DClinPsy^3†^& Konrad Jacobs, DClinPsy^4†^

1. Department of Psychiatry, Warneford Hospital, University of Oxford, Oxford, UK
2. Department of Psychology, University of Oslo, Oslo, Norway
3. City and South Learning Disability Team, Oxford Health NHS Foundation Trust, Oxford, UK
4. Oxford Centre for Children and Young People in Pain, Children’s Hospital, Oxford University Hospitals NHS Foundation Trust, Oxford, UK

† joint senior authors

Corresponding author:

Dr Verena Hinze

University of Oxford, Department of Psychiatry, Warneford Lane, OX3 7JX Oxford, UK

Email: verena.hinze@psych.ox.ac.uk

**Table of Contents**

Figure S1. Pain Body Map.	2

Table S1. Spearman’s Rho Correlation Coefficients between Depression and Anxiety Item Scores and Perceived Burdensomeness and Hopelessness, respectively.	3

Table S2. Correlation Coefficients for Relationships between Aspects of the Chronic Pain Experience and Suicidal Distress.	4

Table S3. Participant Characteristics by Gender.	5

Table S4. Unadjusted Univariable Regression Analyses.	6

Table S5. Regression Analyses, Adjusted for Sadness and Fear, using Standardised Estimates.	7

Figure S1. Pain Body Map.

The Adolescent Pediatric Pain Tool (APPT) was used to create three binary variables, depending on the presence or absence of pain in the following locations:

*Headache (n=44, 57%):* top of head (n=37, 48%), face (n=17, 22%), and back of head (n=22, 29%).

*Abdominal/pelvic pain (n=21, 27%):* abdomen (n=20, 26%) and pelvis (n=3, 4%).

*Musculoskeletal pain (n=74, 96%):* front neck (n=22, 29%), back neck (n=32, 42%), arms/shoulders (n=34, 44%), upper back (n=24, 31%), lower back (n=40, 52%), chest/ribs (n=18, 23%), buttocks (n=11, 14%), hands/wrists (n=31, 40%), legs/hips/knees (n=48, 62%), and feet/ankles (n=39, 51%).

Table S1. Spearman’s Rho Correlation Coefficients between Depression and Anxiety Item Scores and Perceived Burdensomeness and Hopelessness, respectively.

|  | **Perceived Burdensomeness (INQ)** | |  | **Hopelessness (HSC)** | |
| --- | --- | --- | --- | --- | --- |
|  | **r [95% CIs]** | **p-value** |  | **r [95% CIs]** | **p-value** |
| **Depression (BAPQ)** | 0.60 [0.44, 0.73] | **< 0.001** |  | 0.68 [0.52, 0.79] | **< 0.001** |
| I feel sad | 0.47 [0.27, 0.63] | **< 0.001** |  | 0.53 [0.34, 0.66] | **< 0.001** |
| I feel hopeless about the future | 0.54 [0.36, 0.67] | **< 0.001** |  | 0.72 [0.57, 0.81] | **< 0.001** |
| I find it hard to concentrate | 0.30 [0.08, 0.48] | **0.009** |  | 0.39 [0.17, 0.58] | **< 0.001** |
| I feel discouraged | 0.45 [0.25, 0.61] | **< 0.001** |  | 0.53 [0.34, 0.68] | **< 0.001** |
| I think about myself in a negative way | 0.65 [0.47, 0.76] | **< 0.001** |  | 0.60 [0.40, 0.73] | **< 0.001** |
| I feel that everything I do is an effort | 0.38 [0.17, 0.55] | **0.001** |  | 0.43 [0.21, 0.60] | **< 0.001** |
| **Anxiety (BAPQ)** | 0.58 [0.40, 0.71] | **< 0.001** |  | 0.67 [0.52, 0.78] | **< 0.001** |
| I worry about the future | 0.43 [0.22, 0.59] | **< 0.001** |  | 0.56 [0.38, 0.70] | **< 0.001** |
| I feel nervous | 0.48 [0.26, 0.64] | **< 0.001** |  | 0.57 [0.36, 0.71] | **< 0.001** |
| I have feelings of panic | 0.51 [0.30, 0.67] | **< 0.001** |  | 0.60 [0.39, 0.74] | **< 0.001** |
| I feel at ease | -0.21 [-0.41, 0.02] | 0.071 |  | -0.47 [-0.64, -0.22] | **< 0.001** |
| I feel shaky | 0.35 [0.12, 0.54] | **0.002** |  | 0.33 [0.11, 0.50] | **0.004** |
| I feel physically tense | 0.37 [0.16, 0.54] | **0.001** |  | 0.32 [0.10, 0.50] | **0.005** |
| I am afraid | 0.41 [0.20, 0.58] | **< 0.001** |  | 0.46 [0.24, 0.65] | **< 0.001** |

Note. Confidence intervals were estimated, using bias-corrected and accelerated (BCA) bootstrapping (N = 10,000 samples) to account for skewness in the distribution. Highlighted are the items we chose as a measure of sadness and fear, respectively. Strong correlations between the item ‘I feel sad’ and the full depression subscale (r [95%CIs]= 0.73 [0.57, 0.82]) and the item ‘I am afraid’ and the full anxiety subscale (r [95%CIs]=0.73 [0.57, 0.84]) support this choice. INQ = Interpersonal Needs Questionnaire; HSC = Hopelessness Scale for Children; BAPQ = Bath Adolescent Pain Questionnaire.

Table S2. Correlation Coefficients for Relationships between Aspects of the Chronic Pain Experience and Suicidal Distress.

|  | **Perceived Burdensomeness** | |  | **Hopelessness** | |
| --- | --- | --- | --- | --- | --- |
| **Aspects of the chronic pain experience** | **r [95% CIs]** | **p-value** |  | **r [95% CIs]** | **p-value** |
| Pain intensity | 0.04 [-0.19, 0.26] | 0.734 |  | 0.12 [-0.12, 0.35] | 0.303 |
| Number of pain locations | 0.11 [-0.12, 0.33] | 0.349 |  | 0.27 [0.03, 0.46] | **0.020** |
| % sensory words | 0.21 [-0.02, 0.42] | 0.068 |  | 0.27 [0.04, 0.47] | **0.018** |
| % affective words | 0.24 [0.01, 0.45] | **0.035** |  | 0.27 [0.04, 0.48] | **0.016** |
| % evaluative words | 0.18 [-0.04, 0.39] | 0.109 |  | 0.23 [0.00, 0.45] | **0.040** |
| Daily impairment | 0.28 [0.08, 0.47] | **0.013** |  | 0.41 [0.19, 0.58] | **< 0.001** |
| Physical impairment | 0.09 [-0.13, 0.30] | 0.462 |  | 0.18 [-0.06, 0.40] | 0.119 |
| Social impairment | 0.47 [0.28, 0.62] | **< 0.001** |  | 0.57 [0.40, 0.70] | **< 0.001** |
| Cognitive intrusions | 0.42 [0.21, 0.59] | **< 0.001** |  | 0.48 [0.26, 0.66] | **< 0.001** |
| Sadness | 0.47 [0.27, 0.63] | **< 0.001** |  | 0.53 [0.34, 0.66] | **< 0.001** |
| Fear | 0.41 [0.20, 0.58] | **< 0.001** |  | 0.46 [0.24, 0.65] | **< 0.001** |
| Burdensomeness | n/a | n/a |  | 0.69 [0.54, 0.79] | **< 0.001** |
|  | **Self-harm thoughts (past 2 weeks)** | |  | **Self-harm thoughts (Lifetime)** | |
| **Aspects of the chronic pain experience** | **r [95% CIs]** | **p-value** |  | **r [95% CIs]** | **p-value** |
| Pain intensity | -0.06 [-0.28, 0.18] | 0.632 |  | -0.23 [-0.42, -0.01] | **0.049** |
| Number of pain locations | 0.01 [-0.21, 0.26] | 0.911 |  | 0.15 [-0.09, 0.35] | 0.198 |
| % sensory words | 0.18 [-0.04, 0.41] | 0.110 |  | 0.15 [-0.08, 0.33] | 0.206 |
| % affective words | 0.18 [-0.07, 0.44] | 0.120 |  | 0.23 [0.02, 0.39] | **0.045** |
| % evaluative words | 0.15 [-0.08, 0.37] | 0.193 |  | 0.22 [-0.01, 0.42] | 0.058 |
| Daily impairment | 0.05 [-0.15, 0.26] | 0.641 |  | 0.11 [-0.12, 0.32] | 0.342 |
| Physical impairment | -0.08 [-0.26, 0.13] | 0.493 |  | -0.08 [-0.32, 0.15] | 0.484 |
| Social impairment | 0.19 [-0.01, 0.37] | 0.092 |  | 0.29 [0.07, 0.48] | **0.009** |
| Cognitive intrusions | 0.24 [0.03, 0.43] | **0.036** |  | 0.21 [-0.02, 0.41] | 0.069 |
| Sadness | 0.31 [0.13, 0.47] | **0.006** |  | 0.31 [0.08, 0.50] | **0.006** |
| Fear | 0.31 [0.11, 0.49] | **0.006** |  | 0.25 [0.02, 0.45] | **0.030** |
| Burdensomeness | 0.55 [0.33, 0.71] | **< 0.001** |  | 0.43 [0.23, 0.57] | **<0.001** |
| Hopelessness | 0.44 [0.24, 0.61] | **< 0.001** |  | 0.41 [0.19, 0.56] | **<0.001** |
|  | **Self-harm behaviours (past 2 weeks)** | |  | **Self-harm behaviours (Lifetime)** | |
| **Aspects of the chronic pain experience** | **r [95% CIs]** | **p-value** |  | **r [95% CIs]** | **p-value** |
| Pain intensity | 0.09 [-0.11, 0.33] | 0.463 |  | -0.09 [-0.31, 0.14] | 0.438 |
| Number of pain locations | 0.32 [0.08, 0.54] | **0.004** |  | 0.27 [0.05, 0.46] | **0.018** |
| % sensory words | 0.35 [0.08, 0.59] | **0.002** |  | 0.22 [-0.01, 0.41] | 0.053 |
| % affective words | 0.43 [0.04, 0.72] | **<0.001** |  | 0.27 [0.06, 0.44] | **0.016** |
| % evaluative words | 0.25 [0.03, 0.44] | **0.031** |  | 0.26 [0.02, 0.45] | **0.025** |
| Daily impairment | 0.28 [0.14, 0.47] | **0.014** |  | 0.20 [-0.03, 0.40] | 0.081 |
| Physical impairment | 0.20 [0.09, 0.37] | 0.089 |  | 0.02 [-0.21, 0.24] | 0.872 |
| Social impairment | 0.26 [0.14, 0.43] | **0.023** |  | 0.33 [0.12, 0.50] | **0.003** |
| Cognitive intrusions | 0.27 [0.08, 0.49] | 0.017 |  | 0.24 [0.01, 0.43] | **0.039** |
| Sadness | 0.21 [0.10, 0.42] | 0.062 |  | 0.34 [0.13, 0.51] | **0.003** |
| Fear | 0.12 [-0.11, 0.33] | 0.322 |  | 0.31 [0.10, 0.50] | **0.005** |
| Burdensomeness | 0.31 [-0.01, 0.53] | **0.007** |  | 0.37 [0.14, 0.55] | **0.001** |
| Hopelessness | 0.30 [0.07, 0.55] | **0.008** |  | 0.38 [0.16, 0.56] | **<0.001** |

Note. Spearman’s rho correlation coefficients were used for perceived burdensomeness and hopelessness, and point-biserial correlation coefficients were used for self-harm thoughts and behaviours. Confidence intervals were estimated, using bias-corrected and accelerated (BCA) bootstrapping (N=10,000 samples) to account for skewness in the distribution. Significant associations are highlighted in **bold**.

Table S3. Participant Characteristics by Gender.

|  | **Female**  **(n=61; 79%)** | **Male**  **(n=10; 13%)** | **Other**  **(n=6; 8%)** |
| --- | --- | --- | --- |
| **Demographics and mental health covariates** |  |  |  |
| Age in years, mean (SD) [range] | 15.4 (1.5) [12-18 years] | 15.0 (1.63) [12-17 years] | 16.2 (1.47) [14-18 years] |
| Sadness, mean (SD) [range] | 2.52 (0.87) [0-4] | 2.50 (0.53) [2-3] | 2.83 (0.41) [2-3] |
| Fear, mean (SD) [range] | 2.10 (1.19) [0-4] | 2.10 (0.74) [1-3] | 2.17 (0.75) [1-3] |
| **Aspects of the pain experience** |  |  |  |
| Pain intensity, mean (SD) [range] | 3.54 (0.81) [2-5] | 3.50 (0.71) [2-4] | 3.67 (0.52) [3-4] |
| Number of pain locations, mean (SD) [range] | 5.16 (3.15) [1-15] | 3.70 (3.27) [1-11] | 7.67 (3.78) [3-11] |
| Qualitative aspects, mean (SD) [range] |  |  |  |
| Proportion of sensory words | 0.28 (0.17) [0.03-0.73] | 0.16 (0.10) [0.05-0.41] | 0.37 (0.26) [0.11-0.68] |
| Proportion of affective words | 0.21 (0.25) [0.00-1.00] | 0.05 (0.12) [0.00-0.36] | 0.32 (0.33) [0.00-0.91] |
| Proportion of evaluative words | 0.57 (0.30) [0.00-1.00] | 0.34 (0.23) [0.12-0.75] | 0.56 (0.34) [0.25-1.00] |
| Proportion of temporal words | 0.31 (0.15) [0.00-0.82] | 0.27 (0.15) [0.09-0.55] | 0.26 (0.16) [0.09-0.55] |
| Daily impairment, mean (SD) [range] | 33.66 (11.24) [12-56] | 29.9 (11.29) [11-48] | 39.83 (6.49) [30-45] |
| Physical, mean (SD) [range] | 15.26 (7.79) [3-33] | 11.4 (7.57) [4-26] | 18.17 (5.0) [12-26] |
| Social, mean (SD) [range] | 18.39 (6.52) [4-34] | 18.5 (5.8) [5-27] | 21.67 (3.08) [18-25] |
| Cognitive intrusions, mean (SD) [range] | 33.84 (14.68) [0-60] | 25.7 (14.86) [4-50] | 38.00 (6.69) [33-51] |
| **Suicidal distress** |  |  |  |
| Perceived burdensomeness, mean (SD) [range] | 13.00 (8.60) [5-34] | 7.8 (3.91) [5-17] | 21.17 (12.67) [5-32] |
| Hopelessness, mean (SD) [range] | 6.59 (3.65) [1-15] | 4.7 (2.67) [1-11] | 8.17 (5.71) [1-15] |
| Self-harm thoughts, n (%) |  |  |  |
| Recent (past 2 weeks) | 17 (28%) | 1 (10%) | 3 (50%) |
| Lifetime | 35 (57%) | 5 (50%) | 5 (83%) |
| Self-harm behaviours, n (%) |  |  |  |
| Recent (past 2 weeks) | 3 (5%)^a^ | 0 (0%) | 2 (33%) |
| Lifetime | 28 (46%) | 3 (30%) | 5 (83%) |

Note. a: n=1 missing value.

Possible ranges: Age=12-19 years; Sadness=0-4; Fear=0-4; Pain intensity=1(‘no pain’) to 5 (‘worst possible pain’); Number of pain locations=1-15; Qualitative aspects=0.00-1.00; Daily impairment=0-72; Physical impairment=0-36;

Social impairment=0-36; Cognitive intrusions=0-60; Perceived burdensomeness=5-35; Hopelessness=0-17.

Table S4. Unadjusted Univariable Regression Analyses.

|  | **Perceived**  **Burdensomeness** | | | **Hopelessness** | | | **Self-Harm Thoughts**  **(past 2 weeks) †** | | | **Self-Harm Thoughts**  **(lifetime) †** | | | **Self-Harm Behaviours**  **(past 2 weeks) †** | | | **Self-Harm Behaviours**  **(lifetime) †** | | |
| --- | --- | --- | --- | --- | --- | --- | --- | --- | --- | --- | --- | --- | --- | --- | --- | --- | --- | --- |
| **Factor** | **RR** | **95% CI** | **p** | **RR** | **95% CI** | **p** | **OR** | **95% CI** | **p** | **OR** | **95% CI** | **p** | **OR** | **95% CI** | **p** | **OR** | **95% CI** | **p** |
| **Part A: Model-based estimates** | | | | | | | | | | | | | | | | | | |
| Age | 1.04 | [0.93, 1.15] | 0.502 | 1.01 | [0.93, 1.10] | 0.774 | 1.71 | [1.16, 2.69] | **0.011** | 1.13 | [0.83, 1.54] | 0.433 | 0.89 | [0.49, 1.65] | 0.690 | 1.05 | [0.78, 1.42] | 0.750 |
| Gender: Female | REF | REF | REF | REF | REF | REF | REF | REF | REF | REF | REF | REF | REF | REF | REF | REF | REF | REF |
| Male | 0.60 | [0.40, 0.94] | **0.019** | 0.71 | [0.50, 1.06] | 0.081 | 0.29 | [0.02, 1.71] | 0.254 | 0.74 | [0.19, 2.93] | 0.664 | 0.00 | [0.00, 0.00] | 0.994 | 0.51 | [0.10, 2.00] | 0.354 |
| Other | 1.63 | [0.99, 2.91] | 0.075 | 1.24 | [0.79, 2.06] | 0.377 | 2.59 | [0.44, 15.22] | 0.272 | 3.71 | [0.55, 73.44] | 0.244 | 9.50 | [1.04, 77.18] | **0.032** | 5.89 | [0.88, 116.48] | 0.115 |
| Sadness | 1.53 | [1.29, 1.80] | **<.001** | 1.52 | [1.30, 1.77] | **<.001** | 2.84 | [1.37, 6.67] | **0.009** | 2.40 | [1.29, 4.88] | **0.010** | 3.78 | [1.02, 18.18] | 0.066 | 2.66 | [1.40, 5.58] | **0.005** |
| Fear | 1.28 | [1.12, 1.47] | **<.001** | 1.30 | [1.16, 1.45] | **<.001** | 2.01 | [1.23, 3.51] | **0.009** | 1.62 | [1.05, 2.58] | **0.034** | 1.55 | [0.66, 3.98] | 0.323 | 1.86 | [1.20, 3.02] | **0.008** |
| ***Hypothesised effects*** | | | | | | | | | | | | | | | | | | |
| Pain intensity | 1.06 | [0.87, 1.30] | 0.540 | 1.10 | [0.93, 1.30] | 0.266 | 0.85 | [0.43, 1.64] | 0.627 | 0.53 | [0.27, 0.99] | 0.053 | 1.59 | [0.48, 5.79] | 0.459 | 0.79 | [0.43, 1.42] | 0.433 |
| % affective words | 2.16 | [1.23, 3.97] | **0.013** | 2.11 | [1.31, 3.52] | **0.004** | 4.46 | [0.64, 32.38] | 0.127 | 9.28 | [1.16, 119.45] | 0.055 | 113.4 | [5.80, 4826] | **0.004** | 11.6 | [1.60, 121.6] | **0.024** |
| Daily impairment | 1.02 | [1.00, 1.03] | **0.017** | 1.02 | [1.01, 1.04] | **<.001** | 1.01 | [0.97, 1.06] | 0.636 | 1.02 | [0.98, 1.07] | 0.338 | 1.14 | [1.03, 1.30] | **0.028** | 1.04 | [1.00, 1.09] | 0.083 |
| Physical impact | 1.00 | [0.98, 1.03] | 0.795 | 1.01 | [1.00, 1.03] | 0.127 | 0.98 | [0.91, 1.04] | 0.488 | 0.98 | [0.92, 1.04] | 0.479 | 1.11 | [0.98, 1.27] | 0.107 | 1.00 | [0.95, 1.07] | 0.870 |
| Social impact | 1.05 | [1.03, 1.07] | **<.001** | 1.06 | [1.04, 1.08] | **<.001** | 1.08 | [0.99, 1.19] | 0.096 | 1.11 | [1.03, 1.21] | **0.013** | 1.27 | [1.05, 1.64] | **0.034** | 1.13 | [1.04, 1.24] | **0.006** |
| Cognitive intrusions | 1.02 | [1.01, 1.03] | **<.001** | 1.02 | [1.01, 1.03] | **<.001** | 1.04 | [1.00, 1.08] | **0.041** | 1.03 | [1.00, 1.07] | 0.073 | 1.11 | [1.02, 1.24] | **0.030** | 1.04 | [1.00, 1.07] | **0.043** |
| ***Exploratory analyses*** |  |  |  |  |  |  |  |  |  |  |  |  |  |  |  |  |  |  |
| No of pain locations | 1.04 | [0.99, 1.09] | 0.139 | 1.05 | [1.01, 1.09] | **0.018** | 1.01 | [0.86, 1.17] | 0.909 | 1.10 | [0.96, 1.28] | 0.197 | 1.46 | [1.10, 2.12] | **0.017** | 1.19 | [1.03, 1.39] | **0.022** |
| Localised pain | REF | REF | REF | REF | REF | REF | REF | REF | REF | REF | REF | REF | REF | REF | REF | REF | REF | REF |
| Multiple locations | 1.41 | [0.74, 2.50] | 0.265 | 1.90 | [1.11, 3.08] | **0.013** | 2.94 | [0.40, 60.6] | 0.355 | 0.93 | [0.15, 5.82] | 0.935 | 1.00 | [0.00, Inf] | 0.999 | 0.84 | [0.13, 6.96] | 0.858 |
| Widespread pain | 1.52 | [0.81, 2.62] | 0.162 | 2.09 | [1.24, 3.33] | **0.003** | 1.47 | [0.20, 29.9] | 0.738 | 1.93 | [0.32, 11.60] | 0.452 | 0.00 | [0.00, 0.00] | 0.998 | 2.89 | [0.51, 22.47] | 0.248 |
| Constant & flares | REF | REF | REF | REF | REF | REF | REF | REF | REF | REF | REF | REF | REF | REF | REF | REF | REF | REF |
| Constant pain | 0.60 | [0.40, 0.94] | **0.018** | 0.56 | [0.39, 0.83] | **0.002** | 1.17 | [0.23, 4.95] | 0.840 | 0.96 | [0.23, 4.20] | 0.950 | 2.00 | [0.10, 16.18] | 0.559 | 0.86 | [0.20, 3.54] | 0.828 |
| Pain flares | 0.43 | [0.28, 0.69] | **< .001** | 0.56 | [0.38, 0.85] | **0.004** | 0.00 | [0.00, 0.00] | 0.990 | 2.29 | [0.48, 16.54] | 0.333 | 0.00 | [0.00, 0.00] | 0.994 | 0.64 | [0.12, 2.85] | 0.566 |
| % sensory words | 2.76 | [1.21, 6.53] | **0.017** | 2.72 | [1.35, 5.59] | **0.005** | 9.23 | [0.58,156.2] | 0.114 | 5.77 | [0.42, 102.39] | 0.206 | 949.4 | [7.80, Inf] | **0.010** | 13.4 | [0.99, 231.8] | 0.059 |
| % evaluative words | 1.61 | [1.00, 2.62] | 0.060 | 1.68 | [1.11, 2.53] | **0.015** | 3.07 | [0.58,17.60] | 0.193 | 4.48 | [0.97, 22.70] | 0.060 | 66.9 | [1.67, 21229] | 0.063 | 5.75 | [1.26, 29.03] | **0.028** |
| **Part B: Standardised estimates** | | | | | | | | | | | | | | | | | | |
| Age | 1.05 | [0.90, 1.23] | 0.502 | 1.02 | [0.89, 1.16] | 0.774 | 2.26 | [1.26, 4.49] | **0.011** | 1.20 | [0.76, 1.92] | 0.433 | 0.83 | [0.34, 2.15] | 0.690 | 1.08 | [0.68, 1.71] | 0.750 |
| Gender: Female | REF | REF | REF | REF | REF | REF | REF | REF | REF | REF | REF | REF | REF | REF | REF | REF | REF | REF |
| Male | 0.60 | [0.40, 0.94] | **0.019** | 0.71 | [0.50, 1.06] | 0.081 | 0.29 | [0.02, 1.71] | 0.254 | 0.74 | [0.19, 2.93] | 0.664 | 0.00 | [0.00, 0.00] | 0.994 | 0.51 | [0.10, 2.00] | 0.354 |
| Other | 1.63 | [0.99, 2.91] | 0.075 | 1.24 | [0.79, 2.06] | 0.377 | 2.59 | [0.44, 15.22] | 0.272 | 3.71 | [0.55, 73.44] | 0.244 | 9.50 | [1.04, 77.18] | **0.032** | 5.89 | [0.88, 116.48] | 0.115 |
| Sadness | 1.40 | [1.23, 1.60] | **<.001** | 1.40 | [1.24, 1.58] | **<.001** | 2.31 | [1.29, 4.60] | **0.009** | 2.02 | [1.22, 3.57] | **0.010** | 2.86 | [1.01, 9.85] | 0.066 | 2.20 | [1.31, 3.98] | **0.005** |
| Fear | 1.32 | [1.13, 1.53] | **<.001** | 1.33 | [1.18, 1.51] | **<.001** | 2.17 | [1.25, 4.02] | **0.009** | 1.70 | [1.06, 2.86] | **0.034** | 1.61 | [0.64, 4.50] | 0.323 | 1.98 | [1.22, 3.40] | **0.008** |
| ***Hypothesised effects*** | | | | | | | | | | | | | | | | | | |
| Pain intensity | 1.05 | [0.90, 1.22] | 0.540 | 1.08 | [0.95, 1.23] | 0.266 | 0.88 | [0.53, 1.47] | 0.627 | 0.62 | [0.37, 0.99] | 0.053 | 1.43 | [0.57, 3.89] | 0.459 | 0.83 | [0.52, 1.31] | 0.433 |
| % affective words | 1.21 | [1.05, 1.41] | **0.013** | 1.20 | [1.07, 1.37] | **0.004** | 1.45 | [0.89, 2.37] | 0.127 | 1.74 | [1.04, 3.29] | 0.055 | 3.25 | [1.55, 8.28] | **0.004** | 1.84 | [1.12, 3.30] | **0.024** |
| Daily impairment | 1.20 | [1.03, 1.40] | **0.017** | 1.30 | [1.14, 1.47] | **<.001** | 1.13 | [0.68, 1.90] | 0.636 | 1.25 | [0.79, 2.02] | 0.338 | 4.11 | [1.36, 18.09] | **0.028** | 1.52 | [0.96, 2.49] | 0.083 |
| Physical impact | 1.02 | [0.87, 1.21] | 0.795 | 1.11 | [0.96, 1.27] | 0.127 | 0.83 | [0.48, 1.38] | 0.488 | 0.85 | [0.53, 1.34] | 0.479 | 2.17 | [0.88, 6.20] | 0.107 | 1.04 | [0.66, 1.64] | 0.870 |
| Social impact | 1.36 | [1.18, 1.57] | **<.001** | 1.44 | [1.28, 1.61] | **<.001** | 1.60 | [0.94, 2.90] | 0.096 | 1.90 | [1.17, 3.24] | **0.013** | 4.24 | [1.31, 20.98] | **0.034** | 2.12 | [1.28, 3.77] | **0.006** |
| Cognitive intrusions | 1.38 | [1.19, 1.60] | **<.001** | 1.36 | [1.19, 1.55] | **<.001** | 1.79 | [1.05, 3.25] | **0.041** | 1.55 | [0.97, 2.56] | 0.073 | 4.40 | [1.36, 21.49] | **0.030** | 1.65 | [1.03, 2.75] | **0.043** |
| ***Exploratory analyses*** |  |  |  |  |  |  |  |  |  |  |  |  |  |  |  |  |  |  |
| No of pain locations | 1.12 | [0.96, 1.32] | 0.139 | 1.17 | [1.02, 1.34] | **0.018** | 1.03 | [0.61, 1.69] | 0.909 | 1.37 | [0.86, 2.26] | 0.197 | 3.46 | [1.38, 11.78] | **0.017** | 1.78 | [1.11, 2.98] | **0.022** |
| Localised pain | REF | REF | REF | REF | REF | REF | REF | REF | REF | REF | REF | REF | REF | REF | REF | REF | REF | REF |
| Multiple locations | 1.41 | [0.74, 2.50] | 0.265 | 1.90 | [1.11, 3.08] | **0.013** | 2.94 | [0.40, 60.64] | 0.355 | 0.93 | [0.15, 5.82] | 0.935 | 1.00 | [0.00, Inf] | 0.999 | 0.84 | [0.13, 6.96] | 0.858 |
| Widespread pain | 1.52 | [0.81, 2.62] | 0.162 | 2.09 | [1.24, 3.33] | **0.003** | 1.47 | [0.20, 29.90] | 0.738 | 1.93 | [0.32, 11.60] | 0.452 | 0.00 | [0.00, 0.00] | 0.998 | 2.89 | [0.51, 22.47] | 0.248 |
| Constant & flares | REF | REF | REF | REF | REF | REF | REF | REF | REF | REF | REF | REF | REF | REF | REF | REF | REF | REF |
| Constant pain | 0.60 | [0.40, 0.94] | **0.018** | 0.56 | [0.39, 0.83] | **0.002** | 1.17 | [0.23, 4.95] | 0.840 | 0.96 | [0.23, 4.20] | 0.950 | 2.00 | [0.10, 16.18] | 0.559 | 0.86 | [0.20, 3.54] | 0.828 |
| Pain flares | 0.43 | [0.28, 0.69] | **< .001** | 0.56 | [0.38, 0.85] | **0.004** | 0.00 | [0.00, 0.00] | 0.990 | 2.29 | [0.48, 16.54] | 0.333 | 0.00 | [0.00, 0.00] | 0.994 | 0.64 | [0.12, 2.85] | 0.566 |
| % sensory words | 1.20 | [1.03, 1.40] | **0.017** | 1.19 | [1.06, 1.36] | **0.005** | 1.49 | [0.91, 2.46] | 0.114 | 1.37 | [0.86, 2.28] | 0.206 | 3.38 | [1.44, 9.91] | **0.010** | 1.59 | [1.00, 2.64] | 0.059 |
| % evaluative words | 1.16 | [1.00, 1.34] | 0.060 | 1.17 | [1.03, 1.33] | **0.015** | 1.41 | [0.85, 2.40] | 0.193 | 1.58 | [0.99, 2.59] | 0.060 | 3.61 | [1.17, 20.92] | 0.063 | 1.71 | [1.07, 2.79] | **0.028** |

Note. All continuous predictors were mean centered. †Due to the small numbers, the confidence intervals are unstable and estimates need to be interpreted with caution. Significant associations are highlighted in **bold**.

Table S5. Regression Analyses, Adjusted for Sadness and Fear, using Standardised Estimates.

|  | **Perceived**  **Burdensomeness** | | | **Hopelessness** | | | **Self-Harm Thoughts**  **(past 2 weeks) †** | | | **Self-Harm Thoughts**  **(lifetime) †** | | | **Self-Harm Behaviours**  **(past 2 weeks) †** | | | **Self-Harm Behaviours**  **(lifetime) †** | | |
| --- | --- | --- | --- | --- | --- | --- | --- | --- | --- | --- | --- | --- | --- | --- | --- | --- | --- | --- |
| **Factor** | **aRR** | **95% CI** | **p** | **aRR** | **95% CI** | **p** | **aOR** | **95% CI** | **p** | **aOR** | **95% CI** | **p** | **aOR** | **95% CI** | **p** | **aOR** | **95% CI** | **p** |
| Age | 1.06 | [0.92, 1.22] | 0.397 | 1.06 | [0.93, 1.20] | 0.371 | 2.74 | [1.45, 5.93] | **0.004** | 1.31 | [0.80, 2.19] | 0.295 | 0.86 | [0.35, 2.13] | 0.731 | 1.16 | [0.71, 1.93] | 0.566 |
| Female | REF | REF | REF | REF | REF | REF | REF | REF | REF | REF | REF | REF | REF | REF | REF | REF | REF | REF |
| Male | 0.66 | [0.46, 0.99] | **0.032** | 0.78 | [0.55, 1.13] | 0.164 | 0.33 | [0.02, 2.07] | 0.314 | 0.73 | [0.18, 2.98] | 0.655 | 0.00 | [0.00, 0.00] | 0.996 | 0.51 | [0.10, 2.14] | 0.379 |
| Other | 1.51 | [0.96, 2.53] | 0.091 | 1.16 | [0.76, 1.87] | 0.519 | 2.52 | [0.41, 15.8] | 0.304 | 2.98 | [0.42, 60.0] | 0.340 | 11.5 | [1.11, 136.2] | **0.034** | 5.15 | [0.73,104.0] | 0.153 |
| ***Hypothesised effects*** | | | | | | | | | | | | | | | | | | |
| Pain intensity | 0.91 | [0.79, 1.05] | 0.180 | 0.95 | [0.83, 1.08] | 0.423 | 0.61 | [0.32, 1.11] | 0.117 | 0.39 | [0.19, 0.71] | **0.005** | 1.20 | [0.45, 3.47] | 0.723 | 0.58 | [0.31, 1.00] | 0.062 |
| % affective words | 1.07 | [0.94, 1.24] | 0.342 | 1.06 | [0.94, 1.21] | 0.360 | 1.02 | [0.57, 1.77] | 0.955 | 1.39 | [0.78, 2.72] | 0.298 | 3.08 | [1.30, 9.23] | **0.018** | 1.40 | [0.81, 2.60] | 0.249 |
| Daily impairment | 1.10 | [0.96, 1.26] | 0.171 | 1.19 | [1.05, 1.34] | **0.006** | 0.82 | [0.45, 1.48] | 0.514 | 1.04 | [0.62, 1.74] | 0.893 | 3.82 | [1.18, 17.66] | **0.044** | 1.25 | [0.74, 2.15] | 0.399 |
| Physical impact | 1.00 | [0.87, 1.15] | 0.992 | 1.06 | [0.93, 1.21] | 0.336 | 0.71 | [0.37, 1.26] | 0.255 | 0.80 | [0.48, 1.29] | 0.366 | 2.39 | [0.86, 7.96] | 0.110 | 0.98 | [0.59, 1.61] | 0.940 |
| Social impact | 1.22 | [1.06, 1.40] | **0.007** | 1.28 | [1.13, 1.45] | **< .001** | 1.12 | [0.57, 2.21] | 0.732 | 1.54 | [0.89, 2.78] | 0.135 | 4.31 | [1.14, 22.97] | **0.049** | 1.66 | [0.94, 3.08] | 0.090 |
| Cognitive intrusions | 1.22 | [1.04, 1.43] | **0.011** | 1.23 | [1.07, 1.40] | **0.003** | 1.22 | [0.63, 2.39] | 0.557 | 1.15 | [0.63, 2.07] | 0.644 | 4.65 | [1.09, 35.10] | 0.077 | 1.14 | [0.63, 2.07] | 0.660 |
| ***Exploratory analyses*** |  |  |  |  |  |  |  |  |  |  |  |  |  |  |  |  |  |  |
| No of pain locations | 1.05 | [0.91, 1.22] | 0.462 | 1.13 | [1.00, 1.28] | **0.041** | 0.91 | [0.52, 1.54] | 0.719 | 1.28 | [0.78, 2.17] | 0.347 | 3.44 | [1.31, 12.39] | **0.025** | 1.75 | [1.04, 3.09] | **0.041** |
| Localised pain | REF | REF | REF | REF | REF | REF | REF | REF | REF | REF | REF | REF | REF | REF | REF | REF | REF | REF |
| Multiple locations | 1.19 | [0.68, 1.97] | 0.519 | 1.77 | [1.10, 2.73] | **0.014** | 2.95 | [0.32, 65.9] | 0.384 | 0.72 | [0.09, 5.19] | 0.739 | 0.78 | [0.00, Inf] | 0.999 | 0.64 | [0.08, 6.15] | 0.679 |
| Widespread pain | 1.28 | [0.73, 2.08] | 0.352 | 1.90 | [1.19, 2.90] | **0.004** | 1.16 | [0.13, 25.4] | 0.906 | 1.52 | [0.21, 10.6] | 0.667 | 0.00 | [0.00, 0.00] | 0.998 | 2.82 | [0.38, 25.99] | 0.316 |
| Constant & flares | REF | REF | REF | REF | REF | REF | REF | REF | REF | REF | REF | REF | REF | REF | REF | REF | REF | REF |
| Constant pain | 0.79 | [0.52, 1.23] | 0.244 | 0.66 | [0.47, 0.97] | **0.026** | 1.84 | [0.32, 9.63] | 0.465 | 1.53 | [0.32, 8.40] | 0.602 | 2.97 | [0.13, 29.83] | 0.385 | 1.29 | [0.26, 6.48] | 0.752 |
| Pain flares | 0.56 | [0.37, 0.90] | **0.010** | 0.70 | [0.49, 1.05] | 0.071 | 0.00 | [0.00, 0.00] | 0.993 | 5.28 | [0.90, 50.7] | 0.093 | 0.00 | [0.00, 0.00] | 0.994 | 1.09 | [0.19, 5.88] | 0.915 |
| % sensory words | 1.10 | [0.96, 1.26] | 0.192 | 1.13 | [1.00, 1.27] | **0.046** | 1.26 | [0.73, 2.15] | 0.392 | 1.22 | [0.74, 2.09] | 0.437 | 3.43 | [1.34, 11.68] | **0.019** | 1.41 | [0.86, 2.41] | 0.183 |
| % evaluative words | 0.99 | [0.86, 1.15] | 0.924 | 1.01 | [0.89, 1.15] | 0.884 | 0.99 | [0.54, 1.79] | 0.967 | 1.24 | [0.73, 2.11] | 0.428 | 3.08 | [0.88, 19.88] | 0.129 | 1.30 | [0.77, 2.21] | 0.325 |

Note. All continuous predictors were mean centered. †Due to the small numbers, the confidence intervals are unstable and estimates need to be interpreted with caution. Significant associations are highlighted in **bold**.
